# Carbon stable isotopes of glucose during the degradation of rice by the koji fungus *Aspergillus oryzae*

**DOI:** 10.1016/j.heliyon.2024.e33664

**Published:** 2024-06-26

**Authors:** Fumikazu Akamatsu, Ken Oda, Akiko Fujita, Yukari Igi, Atsuko Isogai

**Affiliations:** National Research Institute of Brewing, 3-7-1 Kagamiyama, Higashi-Hiroshima, Hiroshima, 739-0046, Japan

**Keywords:** Amazake, Carbon stable isotope analysis, Hydrolytic enzyme, Filamentous fungus, Monosaccharaide, Saccharification

## Abstract

Glucose, a key component of traditional Japanese fermented foods, is derived from rice starch via saccharification by hydrolytic enzymes produced by *Aspergillus oryzae*. The *δ*^13^C value of glucose reflects that of its rice source. However, the influence of saccharification parameters (glucose concentration, degradation temperature, and reaction time) on glucose *δ*^13^C values is unclear. Here, we investigated the influence of saccharification on the *δ*^13^C value of glucose. Our experiments showed a significant difference in the *δ*^1^³C value of glucose (−27.0 ± 0.1 ‰) obtained from saccharification compared to the ingredient rice (−27.1 ± 0.1 ‰) and remaining solid residue (−27.1 ± 0.1 ‰); however, it did not differ significantly from those of rice koji (−27.0 ± 0.1 ‰) and steamed rice (−27.1 ± 0.1 ‰), despite all values being within 0.1 ‰. Notably, glucose concentration, degradation temperature, and reaction time did not significantly affect glucose *δ*^13^C values. These findings demonstrate the remarkable preservation of glucose *δ*^13^C values. The *δ*^13^C values remain aligned with the original *δ*^13^C value of the rice, even with up to 60 % degradation during *A. oryzae* saccharification. This persistence of the *δ*^13^C value throughout the process offers a potential tool for authenticating the origin of rice-fermented beverages based on the *δ*^13^C value of their glucose component.

## Highlights

• Steamed rice was saccharified by enzymes from the koji fungus *Aspergillus oryzae.*
• Glucose concentrations increased with increasing rice degradation rate.
• The *δ*^13^C values of glucose and rice changed little during saccharification.
• The *δ*^13^C relationship between rice and glucose aids authenticity verification.

## 1 Introduction

Saccharification mediated by the filamentous fungus *Aspergillus oryzae* is a cornerstone process in the production of traditional East Asian fermented foods [[1], [2], [3], [4], [5]]. During koji production, *A. oryzae* grows on steamed rice (*Oryaze sativa*) and secretes hydrolytic enzymes, including α-amylase and glucoamylase, that degrade starch into fermentable sugars [6,7]. This process is essential for generating the glucose required for making numerous traditional Japanese fermented foods, such as sake [8,9], sparkling sake [10], amazake [11,12], shochu [13], vinegar [14], mirin [15,16], miso [17,18], and shoyu [[19], [20], [21]]. The saccharification of rice starch into glucose is critical in sake production, as this sugar is the primary carbon source for sake yeast (*Saccharomyces cerevisiae*), which converts the glucose into ethanol via alcoholic fermentation [22,23]. Therefore, understanding carbon dynamics during saccharification is crucial for optimizing the quality of the final product in traditional fermented food production.

The carbon stable isotopic composition (expressed as *δ*^13^C) of glucose has potential as an indicator of carbon dynamics and as a tool for authenticating the origin of Japanese fermented foods. Previous studies have investigated the utility of *δ*^13^C values in sake produced via simultaneous saccharification and fermentation (SSF) processes [[24], [25], [26]]. However, the variation in the *δ*^13^C value of glucose generated solely from rice starch by *A. oryzae*-derived hydrolytic enzymes remains unknown. Understanding the relationship between the rice grain *δ*^13^C value and that of the resulting glucose would provide key information on the saccharification process employed in traditional fermented rice food production.

This study aimed to quantify carbon isotope discrimination from rice to glucose during the saccharification of rice koji, a process identical to that used for koji amazake, a sweet non-alcoholic fermented beverage made with rice koji and water [11,12,27]. We hypothesized that the *δ*^13^C value of glucose might differ from that of the rice source, with the difference being controlled by the degradation rate. Conversely, we predicted that there would be no difference in the *δ*^13^C values of the rice koji and residual solids remaining after saccharification, compared with the initial rice. This investigation sheds light on variations in glucose *δ*^13^C values during saccharification, offering valuable information derived from carbon stable isotope analysis for the production and evaluation of fermented rice foods.

## 2 Materials and methods

### 2.1 Reagents and materials

d(+)-Glucose (CAS number 50-99-7, 98 %) and d(+)-maltose monohydrate (CAS number 6363-53-7, 98 %) were obtained from Kanto Chemical (Tokyo, Japan). Isomaltose (CAS Number 499-40-1, 98 %) was purchased from Hayashibara (Okayama, Japan). Granular magnesium perchlorate (CAS Number 0034-81-8, 83 %) was purchased from FUJIFILM Wako Pure Chemical Corporation (Osaka, Japan). Chromium oxide (Ⅲ), silvered cobalt oxide (Ⅱ, Ⅲ), and elemental copper (CAS Number 7440-50-8) were obtained from SÄNTIS Analytical AG (Teufen, Switzerland). All reagents used in this study were analytical grade. All aqueous solutions were produced using pure water obtained using a water purification system (Purelite, Organo Corporation, Tokyo, Japan). α-Amylase assay and glucose formation activity kits were purchased from Kikkoman Corporation (Noda, Japan). Industrial filamentous fungus, *Aspergillus oryzae* strain RIBOIS01, stored at the National Research Institute of Brewing (Higashi-Hiroshima, Japan), was used in this study.

### 2.2 Rice koji

The outer layer of the seeds of japonica rice, *Oryza sativa* cultivar Yamadanishiki, was removed using a polishing process, resulting in a 30 % decrease in grain mass. The remaining 70 % (wt/wt) was used as the ingredient rice for rice koji production. The filamentous fungus *A. oryzae* strain RIBOIS01 was cultured on potato dextrose agar plates at 30 °C for 10 days to obtain a conidiospore powder. Rice (1.2 kg) was soaked in water to a moisture content of approximately 130 % (wt/wt), and then steamed for 60 min to an initial moisture content of 130 % (wt/wt). The steamed rice was inoculated with 1.0 × 10^6^ conidiospores/g steamed rice. Aliquots of the inoculated rice (65 g) were placed in plastic containers and cultured in an incubator (KCL2000, Tokyo Rikakikai, Tokyo, Japan) at 30 °C for 24 h. The temperature was the increased from 30 to 40 °C during the period between 24 and 36 h, and maintained at 40 °C for up to 48 h under 95 % relative humidity and mixed every 8 h. This process resulted in complete coverage of the rice by the filamentous fungus. The product was subsequently used as rice koji.

### 2.3 Saccharification

Rice koji (15.024 ± 0.141 g, mean ± standard deviation, *n* = 50) was weighed and added to 50 mL centrifuge tubes with tight caps (VIOLAMO, AS ONE, Osaka, Japan). Pure water (29.994 ± 0.034 g, adjusted to 15 or 50 °C, *n* = 50) was added to each tube, reflecting temperatures commonly used for amazake and sake production, respectively. The mixtures were saccharified at the designated temperatures (15 or 50 °C) for 0, 2, 4, 8, and 24 h (five replicates per time point) to degrade the rice starch by *A. oryzae*-derived hydrolytic enzymes. After saccharification, the liquid was separated from the resulting saccharified rice koji by centrifugation (12,000×*g*, 30 min, 4 °C). Both the liquid and wet rice koji were weighed. The liquid was filtered through a 0.20 μm membrane (VH020P, ADVANTEC, Tokyo, Japan) for further analysis. The wet rice koji was freeze-dried, weighed, and considered as solid residue. The moisture content of the rice koji before saccharification was determined by freeze-drying separate samples to a constant weight (19.74 ± 0.57 %, *n* = 5). The dry weights of the rice koji samples used in the saccharification experiment were calculated by multiplying the wet weight (mg) by the conversion factor 0.8026 obtained in the present study.

### 2.4 Enzyme activity assays

Enzymes were extracted from rice koji according to an official method [28]. The activities of α-amylase and glucoamylase in rice koji (*n* = 5) were determined using an α-amylase and a glucose formation activity kit (Kikkoman Corporation, Noda, Japan), respectively, using a spectrophotometer (UV-1800, Shimadzu Corporation, Kyoto, Japan), following the manufacturer's instructions. The activities of these enzymes are presented as units according to the official method [28].

### 2.5 Chemical analysis

The concentrations of glucose, maltose, and isomaltose in the starch degradation samples were determined using an HPLC system (Shimadzu Corporation) comprising a pump (LC-20AD), refractive-index detector (RID-10A), auto-injector (SIL-20Ai), column oven (CTO-20A), fraction collector (FRC-10A), and system controller (CBM-20A). A VG50-4E polymer-based column (250 × 4.6 mm, 5 μm; Showa Denko, Tokyo, Japan) was used with aqueous acetonitrile (acetonitrile:water 4:1, v/v) as the mobile phase. Each sample was diluted with acetonitrile (acetonitrile:sample 1:1, v/v), filtered through a 0.45 μm membrane, and a 1 μL aliquot was injected into the HPLC system. Elution was conducted at a flow rate of 0.5 mL min^−1^ at 50 °C.

### 2.6 Separation of glucose

Glucose was separated from the samples following Akamatsu et al. [29], using cation-exchange (1 mL Dionex OnGurad II H) and anion-exchange (1 mL Dionex OnGurad II A, Thermo Fisher Scientific, Waltham, MA, USA) solid-phase extraction cartridges to remove amino acids and organic acids to prevent contamination of the columns during HPLC purification. The cartridges were connected in this order, and a 5 mL syringe was attached on top of the cation-exchange cartridge. After conditioning with ultrapure water (15 mL), a sample (1 mL) was passed through the cartridges, followed by washing with ultrapure water (9 mL). The water eluate was freeze-dried for more than 24 h, and then ultrapure water (330 μL) was added and the solution was mixed with acetonitrile (330 μL). Glucose was purified from the eluate containing isomaltose using the Shimadzu HPLC system and the conditions described in Section 2.4. Each sample was filtered through a 0.45 μm membrane, and a 17 μL aliquot was injected into the HPLC system. The HPLC eluate fractions containing glucose were diluted with ultrapure water (1.0 mL) and freeze-dried for 16 h after the removal of acetonitrile by nitrogen blow-drying in a dry bath for 6 min at 40 °C. The dried residue was dissolved in ultrapure water to a final volume of 50 μL.

### 2.7 Carbon stable isotope analysis

Ingredient rice, steamed rice, conidia of *A. oryzae*, koji rice, and solid residue were frozen at −30 °C, freeze-dried to constant weight, and ground to a powder using a mortar and pestle. Aliquots of each sample (0.197 ± 0.012 mg) were placed in tin capsules (5 mm × 9 mm, LUDI SWISS AG, Flawil, Switzerland). Isolated glucose samples (20.0 ± 0.1 μL) were then freeze-dried again in separate tin capsules (5 mm × 9 mm, LUDI SWISS AG). Samples in tin capsules were analyzed in duplicate for carbon stable isotopic composition using a continuous flow system isotope ratio mass spectrometry (IRMS) system. The IRMS system consisted of an elemental analyzer (Flash 2000, Thermo Fisher Scientific) coupled via a Conflo IV interface to an isotope ratio mass spectrometer (Delta V Advantage, Thermo Fisher Scientific). In the elemental analyzer, each sample underwent quantitative combustion with oxygen added to the ultrahigh-purity (UHP) helium carrier gas stream (Iwatani Corporation, Osaka, Japan) at 980 °C in a reactor comprising chromium oxide (Ⅲ) and silvered cobalt oxide. Subsequently, NO_x_ gases were reduced to N_2_ at 650 °C with elemental copper. The resulting gases were passed through water traps containing granular magnesium perchlorate and a GC column for CO_2_ separation, carried by a stream of helium (100 mL min^−1^). Separated CO_2_ was then introduced into the isotope ratio mass spectrometer for carbon stable isotope analysis. The results are reported in delta notation (*δ*) per mil (‰) relative to the Vienna Pee Dee Belemnite (VPDB) as *δ*^13^C = (^13^C/^12^C)_sample_/(^13^C/^12^C)_standard_ − 1, using the terminology of the Commission on Isotopic Abundances and Atomic Weights of the International Union of Pure and Applied Chemistry [30]. Three-point linear calibration using reference materials with known *δ*^13^C values (alanine −19.9 ± 0.2 ‰, glycine −33.8 ± 0.2 ‰, and histidine −11.4 ± 0.2 ‰; Shoko Scientific, Yokohama, Japan) calibrated against international reference materials NBS19 and IAEA–CH–6 from the International Atomic Energy Agency (IAEA) were used for normalization. Each reference material was analyzed in duplicate after every 10 samples for both normalization and quality control. *δ*^1^³C values for the CO_2_ gas derived from the samples were determined relative to a working gas introduced directly into the mass spectrometer from a high-pressure cylinder (99.9995 % purity; Itochu Enex, Tokyo, Japan) using Isodat 3.0 software (Thermo Fisher Scientific) and the ^17^O-correction algorithm for CO_2_ [31]. The standard uncertainty associated with the measurements did not exceed 0.1 ‰ (1σ) across all analytical runs.

### 2.8 Degradation rate

The degradation rate of rice koji during saccharification was estimated as follows:

Degradation rate = (dry weight of rice koji – dry weight of remaining solid) / dry weight of rice koji

### 2.9 Statistical analysis

The effects of glucose concentration, degradation temperature, and reaction time on the *δ*^13^C values of glucose after saccharification were assessed using a generalized linear model (GLM) with a Gaussian distribution and identity link function. Glucose concentration, degradation temperature, and reaction time were included in the model as fixed factors. The effects of degradation temperature, reaction time, and degradation rate on the glucose concentration after degradation were also assessed using a GLM with a gamma distribution and log link function. Degradation temperature, reaction time, and degradation rate were again included in the model as fixed factors. The variance inflation factor (VIF) was calculated to check for collinearity of the factors. The maximum VIF was 3.67 for all models, indicating that collinearity between the factors would not significantly influence the results of the GLMs. The relationships between glucose concentration and the *δ*^13^C values of glucose were independently assessed by linear regression analyses. The mean *δ*^13^C values of glucose, rice koji, steamed rice, rice, koji fungus, and solid content were compared using Tukey's honestly significant difference test. Analyses were performed using R ver. 4.3.2 [32]. For all tests, an *α* value of 0.05 was considered to indicate statistical significance (*P* < 0.05).

## 3 Results and discussion

**Fig. 1:**
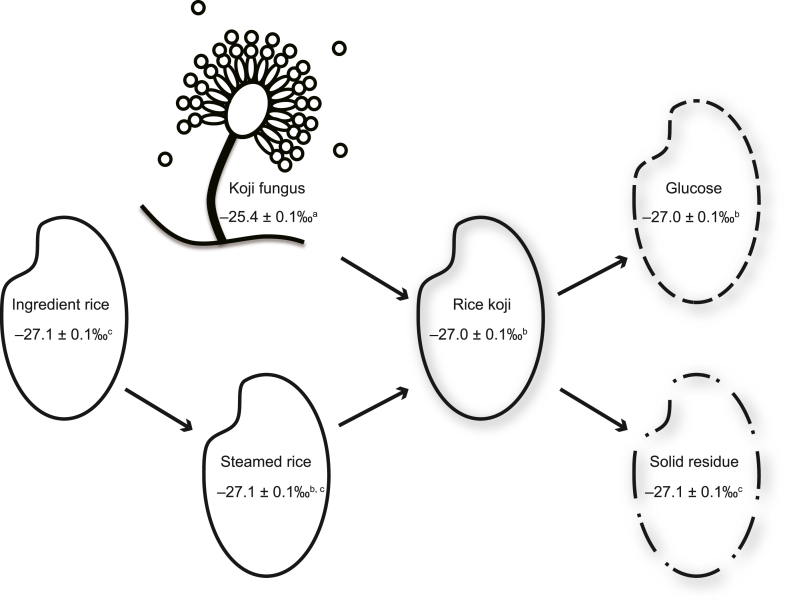
Relationship between the carbon stable isotopic compositions (*δ*^13^C) of glucose (*n* = 50), amazake ingredients (including rice koji, *n* = 10; steamed rice, *n* = 10; ingredient rice, *n* = 10; filamentous fungus, *n* = 10), and by-products (solid residue, *n* = 50) in saccharification experiments. Superscript letters (a, b, and c) denote significant difference (Tukey's honestly significant difference test) in *δ*^13^C value compared with glucose, ingredients, and by-products, respectively.

The *δ*^13^C value of glucose (−27.0 ± 0.1 ‰, mean ± standard deviation) obtained from saccharification differed significantly from those of the ingredient rice (−27.1 ± 0.1 ‰) and remaining solid residue (−27.1 ± 0.1 ‰); however, it did not differ significantly from those of the rice koji (−27.0 ± 0.1 ‰) and steamed rice (−27.1 ± 0.1 ‰), despite all values being within 0.1 ‰ (Fig. 1). Notably, all observed variations in *δ*^13^C values throughout the process, from ingredient rice to glucose production via rice koji, fell within 0.1 ‰ (Fig. 1). This suggests limited carbon isotope discrimination during the degradation of rice starch by hydrolytic enzymes, including α-amylase and glucoamylase. Similarly, no significant differences in *δ*^13^C values were observed between rice koji and steamed rice throughout rice koji preparation, encompassing both the steaming and incubation stages (Fig. 1). This indicates that, on average, carbon isotope discrimination during rice koji preparation is confined to a narrow range of 0.1 ‰. In the post-koji incubation stages, the weight ratio of koji fungus to rice generally remains below 1 % [33]. Therefore, even if the *δ*^13^C values of koji fungus differed from those of rice (Fig. 1), the fungal contribution to the overall *δ*^13^C values would be negligible due to stochiometric limitations. Our findings suggest that carbon isotope discrimination during saccharification and fungal growth associated with *A. oryzae* in rice is confined to a narrow range of 0.1 ‰.

**Fig. 2:**
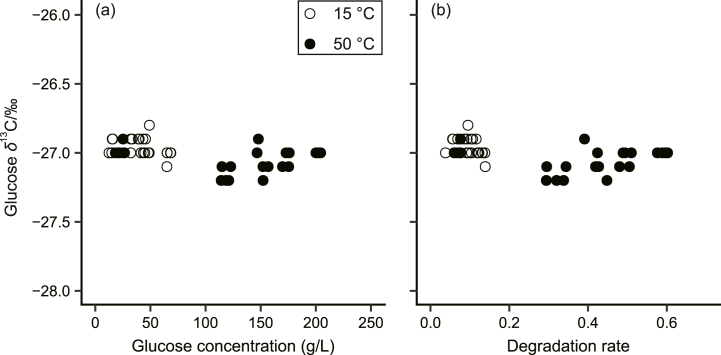
Relationships between (a) the concentration and carbon stable isotopic composition (*δ*^13^C) of glucose, and (b) the degradation rate of rice koji and the *δ*^13^C value of glucose after saccharification experiments at 15 and 50 °C.

The final *δ*^13^C values of glucose were not significantly affected by glucose concentration, degradation temperature, or reaction time (Table 1). This aligns with the observed lack of significant correlation between glucose concentration and *δ*^13^C values, despite a wide range of values being observed following saccharification (Fig. 2a). Notably, the *δ*^13^C values of glucose mirrored those of the original rice, even after substantial degradation of up to 60 % (Fig. 2b). This preservation of *δ*^13^C values throughout saccharification using *A. oryzae*-derived enzymes ensures the authenticity of the final product in beverages such as amazake, where rice conversion to glucose typically involves 5–8 h of degradation at 50 °C [11]. Low-temperature degradation of rice koji (e.g., at 15 °C) is common in sake production. In this case, sake lees typically comprise 45 % of the total ingredient rice weight for premium varieties such as ginjo and daiginjo sake (*n* = 826) [34]. Although the *δ*^13^C values of glucose are retained from the ingredient rice throughout saccharification, the resulting sake after SSF exhibits *δ*^13^C values of glucose that are ∼1 ‰ more positive than that of the ingredient rice [25]. This discrepancy is roughly half of the carbon isotope discrimination observed during the single fermentation step of winemaking, where grape sugar is converted to ethanol [[35], [36], [37]]. These results suggest that sake production can be attributed to the combined effects of: (1) the enrichment of the substrate with isotopically more positive glucose arising from kinetic effects during fermentation, and (2) the incorporation of the original glucose due to limited carbon isotope discrimination during saccharification by *A. oryzae* enzymes, as confirmed by this study.

**Table 1:** Generalized linear model results for testing the effects of glucose concentration, degradation temperature, and reaction time on the carbon stable isotopic composition (*δ*^13^C) of glucose obtained from degraded rice koji after saccharification (*n* = 50).

	Glucose concentration		Degradation temperature		Reaction time	
	*F*	*P*	*F*	*P*	*F*	*P*
Glucose *δ*^13^C	0.71	0.405	2.50	0.121	0.15	0.705

**Fig. 3:**
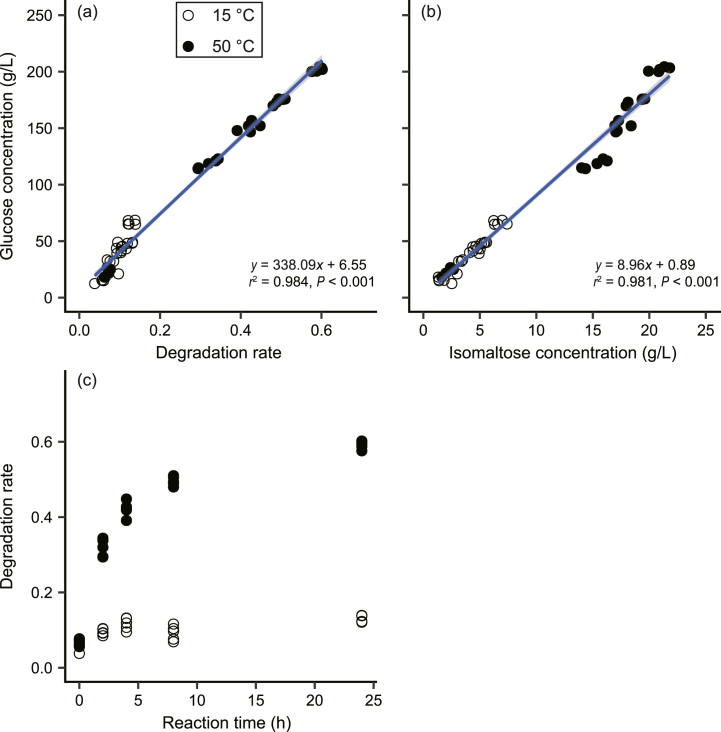
Relationships between (a) the degradation rate of rice koji and glucose concentration, (b) the isomaltose concentration and glucose concentration, and (c) the reaction time and degradation rate of rice koji after saccharification experiments at 15 and 50 °C. Solid lines and shaded regions represent linear regression lines and 95 % confidence intervals, respectively.

A strong positive correlation between the degradation rate of rice koji and glucose concentration confirmed the efficient conversion of rice starch into glucose by *A. oryzae*-derived amylases during saccharification (Fig. 3a). Glucose concentration also exhibited a robust correlation with isomaltose concentrations (Fig. 3b), with glucose being 9.4-fold higher on average, while maltose was not detected. High activities of α-amylase (901.3 ± 43.9 U/g) and glucoamylase (182.1 ± 9.8 U/g) in the rice koji further supported this conversion. The observed higher conversion rate at 50 °C (∼60 % after 24 h) than at 15 °C (∼10 % after 24 h) (Fig. 3c) aligns with the reported optimal activity of α-amylase from *A. oryzae* at ∼50 °C [11,27,38,39], highlighting the importance of temperature optimization for maximizing enzymatic conversion rates. Interestingly, our results suggest that the final post-saccharification glucose concentration is determined mainly by the degradation rate of the rice koji, rather than the specific temperature and reaction time utilized (Table 2). While tailoring the temperature to promote maximum enzyme activity typically achieves optimal saccharification efficiency (e.g., 50 °C for α-amylase and 60 °C for glucoamylase) [[38], [39], [40]], sake production processes often utilize lower temperatures (∼15 °C) for slower, more controlled saccharification achieved through SSF. This finding suggests that, within a reasonable range, the final glucose concentration during saccharification can be estimated from the degradation rate alone, independent of the specific temperature and reaction time.

**Table 2:** Generalized linear model results for testing the effects of degradation rate, degradation temperature, and reaction time on the glucose concentration from degraded rice koji after saccharification (*n* = 50).

	Degradation rate		Degradation temperature		Reaction time	
	*F*	*P*	*F*	*P*	*F*	*P*
Glucose concentration	66.03	<0.001	0.19	0.662	2.76	0.104

## 4 Conclusions

This study reveals that the *δ*^13^C values of glucose produced from rice via *A. oryzae*-mediated saccharification are remarkably preserved relative to the original rice ingredient. Neither glucose concentration, degradation temperature, nor reaction time significantly influenced the final *δ*^13^C values of glucose. Even with up to 60 % starch degradation, carbon isotope discrimination of glucose derived from rice starch during saccharification by *A. oryzae*-produced hydrolytic enzymes remained within 0.1 ‰. This *δ*^13^C preservation offers valuable information for determining the ingredients in traditional processed rice foods using carbon stable isotope analysis. This information can be crucial for ensuring the authenticity and brand enhancement of these products.

## Funding

This research did not receive any specific grant from funding agencies in the public, commercial, or not-for-profit sectors.

## CRediT authorship contribution statement

**Fumikazu Akamatsu:** Writing – original draft, Visualization, Methodology, Investigation, Formal analysis, Conceptualization. **Ken Oda:** Writing – review & editing, Resources, Methodology, Investigation, Conceptualization. **Akiko Fujita:** Methodology, Conceptualization. **Yukari Igi:** Investigation. **Atsuko Isogai:** Writing – review & editing.

## Declaration of competing interest

The authors declare that they have no known competing financial interests or personal relationships that could have appeared to influence the work reported in this paper.

## References

[1] Allwood J.G., Wakeling L.T., Post L.S., Bean D.C. (2023). Food safety considerations in the production of traditional fermented products: Japanese rice koji and miso. J. Food Saf..

[2] Kaewkrod A., Niamsiri N., Likitwattanasade T., Lertsiri S. (2018). Activities of macerating enzymes are useful for selection of soy sauce koji. LWT–Food Sci. Technol..

[3] Saeed F., Afzaal M., Shah Y.S., Khan M.H., Hussain M., Ikram A., Ateeq H., Noman M., Saewan S.A., Khashroum A.O. (2022). Miso: a traditional nutritious & health-endorsing fermented product. Food Sci. Nutr..

[4] Sassi S., Wan-Mohtar W.A.A.Q.I., Jamaludin N.S., Ilham Z. (2021). Recent progress and advances in soy sauce production technologies: a review. J. Food Process. Preserv..

[5] Tamang J.P., Cotter P.D., Endo A., Han N.S., Kort R., Liu S.Q., Mayo B., Westerik N., Hutkins R. (2020). Fermented foods in a global age: East meets West. Compr. Rev. Food Sci. Food Saf..

[6] Daba G.M., Mostafa F.A., Elkhateeb W.A. (2021). The ancient koji mold (*Aspergillus oryzae*) as a modern biotechnological tool. Bioresour. Bioprocess..

[7] Wisman A.P., Tamada Y., Hirohata S., Gomi K., Fukusaki E., Shimma S. (2020). Mapping *haze-komi* on rice *koji* grains using β-glucuronidase expressing *Aspergillus oryzae* and mass spectrometry imaging. J. Biosci. Bioeng..

[8] Kitamoto K. (2015). Cell biology of the *Koji* mold *Aspergillus oryzae*. Biosci. Biotechnol. Biochem..

[9] Negoro H., Ishida H. (2022). Development of sake yeast breeding and analysis of genes related to its various phenotypes. FEMS Yeast Res..

[10] Akamatsu F., Fujii T., Igi Y., Fujita A., Yamada O., Isogai A. (2022). Different carbon stable isotopic compositions of CO_2_ in sparkling sake using natural and exogenous carbonation methods. J. Food Compos. Anal..

[11] Oguro Y., Nakamura A., Kurahashi A. (2019). Effect of temperature on saccharification and oligosaccharide production efficiency in *koji amazake*. J. Biosci. Bioeng..

[12] Saigusa N., Ohba R. (2007). Effects of *koji* production and saccharification time on the antioxidant activity of *amazake*. Food Sci. Technol. Res..

[13] Shiraishi Y., Yoshizaki Y., Ono T., Yamato H., Okutsu K., Tamaki H., Futagami T., Yoshihiro S., Takamine K. (2016). Characteristic odour compounds in *shochu* derived from rice *koji*. J. Inst. Brew..

[14] Suzuki E., Otake S., Hamadate N., Hasumi K. (2020). Kurozu melanoidin, a novel oligoglucan-melanoidin complex from Japanese black vinegar, suppresses adipogenesis *in vitro*. J. Funct.Foods.

[15] Hashizume K., Ito T., Ishizuka T., Takeda N. (2013). Formation of ethyl ferulate by rice *koji* enzyme in sake and mirin mash conditions. J. Biosci. Bioeng..

[16] Kaneko S., Kumazawa K. (2015). Aroma compounds in Japanese sweet rice wine (Mirin) screened by aroma extract dilution analysis (AEDA). Biosci. Biotechnol. Biochem..

[17] Kojo T., Kawai M., Shiraishi Y., Kurazono S., Kadooka C., Okutsu K., Yoshizaki Y., Ikenaga M., Futagami T., Takamine K., Tamaki H. (2019). Effect of maturation time on koji-like smell and volatile compounds of barley miso (Japanese soybean paste) during fermentation. Food Sci. Technol. Res..

[18] Yamabe S., Kobayashi-Hattori K., Kaneko K., Endo H., Takita T. (2007). Effect of soybean varieties on the content and composition of isoflavone in rice-koji miso. Food Chem..

[19] Asanuma K., Wang Z., Miyazaki T., Yuan C., Yamashita T. (2024). Development and characterization of Japanese soy sauce-like fermented seasoning with various ingredients. Food Biosci..

[20] Nishimura I., Shinohara Y., Oguma T., Koyama Y. (2018). Survival strategy of the salt-tolerant lactic acid bacterium, *Tetragenococcus halophilus*, to counteract koji mold, *Aspergillus oryzae*, in soy sauce brewing. Biosci. Biotechnol. Biochem..

[21] Watarai N., Yamamoto N., Sawada K., Yamada T. (2019). Evolution of *Aspergillus oryzae* before and after domestication inferred by large-scale comparative genomic analysis. DNA Res..

[22] Okuda M. (2019). Rice used for Japanese sake making. Biosci. Biotechnol. Biochem..

[23] Watanabe D. (2024). Sake yeast symbiosis with lactic acid bacteria and alcoholic fermentation. Biosci. Biotechnol. Biochem..

[24] Akamatsu F., Hashiguchi T., Igi Y., Izu H., Fujii T. (2017). Carbon stable isotope analysis for glucose in sake: simple freeze-dried sake can substitute for glucose following HPLC isolation. Food Anal. Methods.

[25] Akamatsu F., Igi Y., Fujita A., Yamada O., Okuda M. (2023). Carbon stable isotopic compositions of glucose and ethanol in sake after simultaneous saccharification and fermentation processes. Food Chem..

[26] Suto M., Kawashima H. (2019). Compound specific carbon isotope analysis in sake by LC/IRMS and brewers' alcohol proportion. Sci. Rep..

[27] Santos M.V., Banfi S., Santos R., Mota M., Raymundo A., Prista C. (2023). Improving chestnut physicochemical properties through fermentation – development of chestnut Amazake. Food Chem. X.

[28] National Research Institute of Brewing (2017). https://www.nrib.go.jp/bun/nribanalysis.htm.

[29] Akamatsu F., Igi Y., Fujita A. (2020). Separation and purification of glucose in sake for carbon stable isotope analysis. Food Anal. Methods.

[30] Skrzypek G., Allison C.E., Böhlke J.K., Bontempo L., Brewer P., Camin F., Carter J.F., Chartrand M.M.G., Coplen T.B., Gröning M., Hélie J.-.F., Esquivel-Hernández G., Kraft R.A., Magdas D.A., Mann J.L., Meija J., Meijer H.A.J., Moossen H., Ogrinc N., Perini M., Possolo A., Rogers K.M., Schimmelmann A., Shemesh A., Soto D.X., Thomas F., Wielgosz R., Winchester M.R., Yan Z., Dunn P.J.H. (2022). Minimum requirements for publishing hydrogen, carbon, nitrogen, oxygen and sulfur stable-isotope delta results (IUPAC Technical Report). Pure Appl. Chem..

[31] Santrock J., Studley S.A., Hayes J.M. (1985). Isotopic analyses based on the mass spectra of carbon dioxide. Anal. Chem..

[32] R Core Team (2023). https://www.R-project.org/.

[33] Gomi K., Okazaki N., Tanaka T., Kumagai C., Inoue H., Iimura Y., Hara S. (1987). Estimation of mycelial weight in rice-*koji* with use of fungal cell wall lytic enzyme. J. Brew. Soc. Jpn..

[34] Yamada O., Isogai A., Fujita A., Kishimoto T., Akamatsu F., Boerzhijin S., Nishimoto M., Kanda R., Teramoto S., Ogushi K., Fukuda H. (2023). Analysis of sake components presented to the Japan Sake Awards 2022. Rep. Natl. Res. Inst. Brew..

[35] Akamatsu F., Shimizu H., Igi Y., Kamada A., Koyama K., Yamada O., Goto-Yamamoto N. (2022). Prediction method for determining the carbon stable isotopic composition of berry sugars in the original must of Chardonnay wines. Food Chem..

[36] Roßmann A., Schmid H.-L., Reniro F., Versini G., Moussa I., Merle M.H. (1996). Stable carbon isotope content in ethanol of EC data bank wines from Italy, France and Germany. Z. Lebensm. Unters. Forsch..

[37] Spangenberg J.E., Zufferey V. (2019). Carbon isotope compositions of whole wine, wine solid residue, and wine ethanol, determined by EA/IRMS and GC/C/IRMS, can record the vine water status—a comparative reappraisal. Anal. Bioanal. Chem..

[38] Kundu A.K., Das S. (1970). Production of amylase in liquid culture by a strains of *Aspergillus oryzae*. Appl. Microbiol..

[39] Ramachandran S., Patel A.K., Nampoothiri K.M., Chandran S., Szakacs G., Soccol C.R., Pandey A. (2004). Alpha amylase from a fungal culture grown on oil cakes and its properties. Braz. Arch. Biol. Technol..

[40] Parashar D., Satyanarayana T. (2017). Engineering a chimeric acid-stable α-amylase-glucoamylase (Amy-Glu) for one step starch saccharification. Int. J. Biol. Macromol..

